# Microbial community structure in aquifers associated with arsenic: analysis of 16S rRNA and arsenite oxidase genes

**DOI:** 10.7717/peerj.10653

**Published:** 2021-01-08

**Authors:** Prinpida Sonthiphand, Pasunun Rattanaroongrot, Kasarnchon Mek-yong, Kanthida Kusonmano, Chalida Rangsiwutisak, Pichahpuk Uthaipaisanwong, Srilert Chotpantarat, Teerasit Termsaithong

**Affiliations:** 1 Department of Biology, Faculty of Science, Mahidol University, Bangkok, Thailand; 2 Bioinformatics and Systems Biology Program, School of Bioresources and Technology, King Mongkut’s University of Technology Thonburi, Bangkok, Thailand; 3 Systems Biology and Bioinformatics Research Laboratory, Pilot Plant Development and Training Institute, King Mongkut’s University of Technology Thonburi, Bangkok, Thailand; 4 Department of Geology, Faculty of Science, Chulalongkorn University, Bangkok, Thailand; 5 Research Program on Controls of Hazardous Contaminants in Raw Water Resources for Water Scarcity Resilience, Center of Excellence on Hazardous Substance Management (HSM), Chulalongkorn University, Bangkok, Thailand; 6 Research Unit of Green Mining (GMM), Chulalongkorn University, Bangkok, Thailand; 7 Learning Institute, King Mongkut’s University of Technology Thonburi, Bangkok, Thailand; 8 Theoretical and Computational Science Center (TaCS), King Mongkut’s University of Technology Thonburi, Bangkok, Thailand

**Keywords:** Microbiome, Deep groundwater, Shallow groundwater, AioA gene, Arsenite-oxidizing bacteria, Arsenic, Arsenite oxidase

## Abstract

The microbiomes of deep and shallow aquifers located in an agricultural area, impacted by an old tin mine, were explored to understand spatial variation in microbial community structures and identify environmental factors influencing microbial distribution patterns through the analysis of 16S rRNA and *aioA* genes. Although *Proteobacteria*, *Cyanobacteria*, *Actinobacteria*, *Patescibacteria*, *Bacteroidetes*, and *Epsilonbacteraeota* were widespread across the analyzed aquifers, the dominant taxa found in each aquifer were unique. The co-dominance of *Burkholderiaceae* and *Gallionellaceae* potentially controlled arsenic immobilization in the aquifers. Analysis of the *aioA* gene suggested that arsenite-oxidizing bacteria phylogenetically associated with *Alpha*-, *Beta*-, and *Gamma proteobacteria* were present at low abundance (0.85 to 37.13%) and were more prevalent in shallow aquifers and surface water. The concentrations of dissolved oxygen and total phosphorus significantly governed the microbiomes analyzed in this study, while the combination of NO_3_^-^-N concentration and oxidation-reduction potential significantly influenced the diversity and abundance of arsenite-oxidizing bacteria in the aquifers. The knowledge of microbial community structures and functions in relation to deep and shallow aquifers is required for further development of sustainable aquifer management.

## Introduction

Groundwater ecosystems are important reservoirs, holding 94% of all available freshwater. Not only do groundwater ecosystems provide the main source of drinking water worldwide (Griebler & Avramov, 2015), they also contribute to the recycling of elements (e.g., C, N, and S) and the biodegradation of anthropogenic pollutants (e.g., fertilizers, pesticides, and hydrocarbons) in impacted aquifers Chotpantarat, Parkchai & Wisitthammasri, 2020; Griebler & Avramov, 2015; Jewell et al., 2016; Kotik et al., 2013; Wisitthammasri, Chotpantarat & Thitimakorn, 2020). These two latter services provided by groundwater ecosystems are dependent mainly on the existence and activity of specific microbial taxa. Groundwater ecosystems are energy-limited habitats because of their low oxygen concentrations and the lack of sunlight: however, they harbor much more diverse microbial communities than previously suspected (Griebler & Avramov, 2015; Herrmann et al., 2019; Probst et al., 2018).

Microbiome analysis of the 16S rRNA gene reveals that *Proteobacteria*, *Firmicutes*, *Bacteroidetes*, *Planctomycetes*, *Actinobacteria*, *OD1*, *Verrucomicrobia*, and *Nitrospirae* are common constituent taxa of the groundwater microbiome (Cavalca et al., 2019; Das et al., 2017; Lee, Unno & Ha, 2018; Sonthiphand et al., 2019). However, some specific microbial assemblages occur in groundwater at particularly high abundance. *Candidatus* Kaiserbacteraceae, *Candidatus* Nomurabacteraceae, and unclassified UBA9983, members of the phylum *Patescibacteria*, were highly represented in the shallowest groundwater well (5.1 m depth) of the Hainich Critical Zone Exploratory (CZE) in Germany (Herrmann et al., 2019). These microbial taxa involve in driving the nitrogen, sulfur and iron cycles. *Rhodospirillales*, *Rhodocyclales*, *Chlorobia*, and *Circovirus* were dominant in the shallow groundwater, whereas *Deltaproteobacteria* and *Clostridiales* were predominant in the deep groundwater of the Ashbourne aquifer system in South Australia (Smith et al., 2012). These microorganisms harbor metabolic genes involved in antibiotic resistance, lactose and glucose utilization, flagella production, phosphate metabolism, and starch uptake pathways (Smith et al., 2012). *Candidatus* Altiarchaeum sp. and *Sulfurimonas* respectively dominated in the deep and shallow aquifers of the Paradox Basin in USA (Probst et al., 2018). *Candidatus* Altiarchaeum sp. and *Sulfurimonas* are capable of reducing sulfite with carbon fixation and oxidizing sulfide with N_2_ fixation, respectively (Probst et al., 2018). Unlike groundwater microbiomes, surface water (e.g., lakes and rivers) microbiomes generally host *hgcI clade* and *Limnohabitans*, belonging to the classes *Actinobacteria* and *Betaproteobacteria*, respectively (Keshri, Ram & Sime-Ngando, 2018; Ram, Keshri & Sime-Ngando, 2019). Members of *Limnohabitans* contribute to the carbon flow through food chains as they are able to consume algal derivatives for their growth (Šimek et al., 2010). Members of *hgcI clade* have a competitive advantage over others to survive in energy-limited and nutrient-limited environments (Ghylin et al., 2014). However, previous studies demonstrated the mobilizations of microbial taxa across different biomes (Herrmann et al., 2019; Monard et al., 2016). That said, microorganisms found in one biome are possibly transferred from an adjacent biome, such as from terrestrial to freshwater or from soil to groundwater.

Our study area was located in an intensively agricultural landscape, impacted by an old tin mine, where the arsenic (As) concentration in soils was high, in the range of 4.84–1,070.42 mg kg^−1^ (Tiankao & Chotpantarat, 2018). The arsenic concentration in a particular shallow groundwater well (14 µg l^−1^) located downstream of the old tin mine exceeded the World Health Organization (WHO) limit of 10 µg l^−1^ (Tiankao & Chotpantarat, 2018). Due to its extreme toxicity, As contamination in groundwater is an issue of global environmental concern, which directly affects human health (Boonkaewwan, Sonthiphand & Chotpantarat, 2020; Cavalca et al., 2019; Chotpantarat et al., 2014; Das et al., 2017; Li et al., 2013; Wongsasuluk et al., 2018a; Wongsasuluk et al., 2018b). Previous studies have suggested that microorganisms are responsible for reducing the toxicity, solubility, and mobility of arsenic in impacted aquifers through arsenite oxidation (Li et al., 2016; Osborne et al., 2010). Arsenite oxidation is performed by arsenite-oxidizing bacteria using the key enzyme arsenite oxidase (aio), converting toxic arsenite (As^3+^) to arsenate (As^5+^). Chemolithoautotrophic arsenite-oxidizing bacteria are able to use As^3+^ as an electron donor and use O_2_, NO_3_^−^, or Fe ^3+^ as an electron acceptor for their energy metabolism (Páez-Espino et al., 2009). Both cultured and uncultured arsenite-oxidizing bacteria distributed in various environments have been examined by analysis of the *aioA* gene, encoding a large subunit of arsenite oxidase (aioA). Molecular surveys of the *aioA* gene have recovered arsenite-oxidizing bacteria of the classes *Alphaproteobacteria*, *Betaproteobacteria*, and *Gammaproteobacteria* from aquifers across various locations (Cavalca et al., 2019; Quéméneur et al., 2010).

There is very limited information on the microbial community structures, including the diversity and abundance of arsenite-oxidizing bacteria, in deep and shallow aquifers impacted by the combination of land uses. Due to the unique physicochemical characteristics of deep and shallow aquifers, land uses, and the history of the study area, we hypothesized that the communities of microorganisms and arsenite-oxidizing bacteria in each aquifer were distinct. This study aimed to elucidate the microbial community structures in deep and shallow aquifers and identify environmental factors influencing their distribution patterns using an Illumina MiSeq platform targeting the V3-V4 region of the 16S rRNA gene. In addition, the diversity and abundance of arsenite-oxidizing bacteria in the aquifers were investigated by analysis of the *aioA* gene using PCR-cloning-sequencing and quantitative PCR (qPCR). This study sheds light on spatial variations of microbiomes in relation to deep and shallow aquifers impacted by agricultural and mining activities, and expands knowledge of the diversity and abundance of arsenite-oxidizing bacteria which play a vital role in arsenic bioremediation, especially in aquifers receiving external pollutants (e.g., agricultural and mining activities).

**Figure 1:**
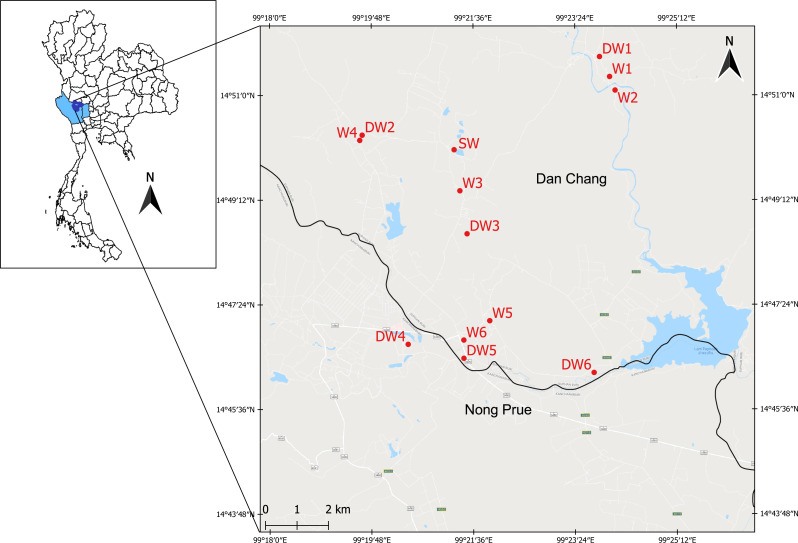
Study area showing the sampling locations of six deep groundwaters (DW1–DW6), six shallow groundwaters (W1–W6), and surface water (SW).

## Materials & Methods

### Sampling site description and sample collection

The study area was located in the Lower Chao Praya Basin, Thailand, in Dan Chang District, Suphan Buri Province, and in adjacent Nong Prue District, Kanchanaburi Province (Fig. 1). The sampling area covered an old tin mine, currently used for agricultural purposes, including sugarcane and corn cultivation. Arsenic concentrations in soils from the old mine within this area were considerably high (52.12–1,070.42 mg kg^−1^) and in one particular shallow groundwater well (14 µg l^−1^) exceeded the maximum admissible concentration of 10 µg l^−1^ set by WHO (Tiankao & Chotpantarat, 2018). Groundwater is commonly used by locals for daily consumption. Mr. Narong Ketprapum, the President of Dan Chang Subdistrict Administrative Organization, and Mr. Surasi Songcharoen, the President of Nong Prue Subdistrict Administrative Organization, gave verbal permission for the collection of water samples. In this study, water samples were collected from three aquifer types: deep groundwater (DW), shallow groundwater (W), and surface water (SW). Twelve groundwater samples and one surface water sample were collected on April 5th and 6th, 2018. All groundwater samples were collected from currently active wells, six deep groundwater wells (DW1 to DW6) and six shallow groundwater wells (W1 to W6) (Fig. S1). To obtain a representative groundwater sample, groundwaters were purged for approximately 10 min before sampling. Deep groundwater samples were directly collected from a tube well using a high density polyethylene (HDPE) plastic container. Shallow groundwater samples were collected from a ring well using a polyethylene bailer. The water table of each of the six shallow wells (W1 to W6), ranging from 3–6 m, were measured onsite using an electric tape. Those of the six deep wells (DW1 to DW6) could not be analyzed due to the limitation of their aquifer structure. The single surface water sample (SW) was collected from an old tailing pond (Fig. 1). The surface water sample was randomly collected from five locations from the pond; it was subsequently pooled on site. All groundwater and surface water samples were collected in triplicate and kept on ice during transportation.

### Physicochemical analyses

Physicochemical parameters of groundwater and surface water samples were analyzed. Oxidation–reduction potential (ORP) and pH were measured on site using a portable pH meter (WTW, USA). Conductivity (EC), dissolved oxygen concentration (DO), and temperature were also measured at the field sites using a Hach meter (Hach, USA). For other physicochemical parameters, all water samples were preserved on-site according to standard protocols (APHA 2012). Total Kjeldahl nitrogen (TKN), nitrate-nitrogen (NO_3_^−^-N), total phosphorus (TP), and total carbon (TC) were respectively analyzed by the macro-Kjeldahl method, cadmium reduction, the ascorbic acid method, and a total organic carbon analyzer (APHA, 2012). The concentrations of total arsenic and As^3+^ were measured using an inductively coupled plasma mass spectrometer (ICP-MS) according to previously published protocols (APHA, 2012). All analyzed water samples were filtered through an arsenate removal cartridge following the manufacturer’s protocol (Meng & Wang, 1998). The cartridge selectively adsorbs As^5+^: the filtered water samples were subsequently collected for the analysis of As^3+^ concentration. The concentration of As^5+^ was calculated from the difference between the concentrations of total arsenic and As^3+^. All analyzed physicochemical parameters of the water samples are shown in Table 1.

**Table 1:** Physicochemical parameters of water samples.

ID	DO (mg l^−1^)	pH	ORP (mV)	EC (µs cm^−1^)	Temp (°C)	TKN (mg l^−1^)	NO_3_^−^-N (mg l^−1^)	TP (mg l^−1^)	TC (mg l^−1^)	Total arsenic (µg l^−1^)	As^3+^ (µg l^−1^)	As^5+^ (µg l^−1^)
DW1	2.14	6.56	−64.8	544	28.8	0.3	<0.05	0.01	51.66	6.35	3.84	2.51
DW2	5.31	6.64	173.7	350	31.5	<0.1	0.11	0.01	29.92	0.59	0.28	0.31
DW3	4.74	6.55	172.6	371	28.5	<0.1	<0.05	0.01	4.965	1.93	1.11	0.83
DW4	2.48	6.42	189.7	362	30.2	<0.1	<0.05	0.01	6.925	2.38	0.36	2.02
DW5	4.39	6.51	144.3	362	30	<0.1	0.07	0.01	1.656	0.60	0.33	0.27
DW6	4.84	6.89	177.5	589	30.6	0.4	<0.05	0.01	37.1	9.13	5.70	3.43
W1	2.77	6.42	147.5	270	25.8	0.4	0.11	0.02	33.77	0.41	0.27	0.13
W2	2.47	6.3	217.2	350	30	0.3	0.12	0.06	31.77	1.48	0.41	1.08
W3	2.49	6.24	87	330	30.6	<0.1	<0.05	0.13	17.77	5.41	0.74	4.67
W4	3.6	6.6	199.4	282	27.4	0.2	<0.05	0.06	6.978	2.85	0.28	2.57
W5	2.5	6.53	208.2	546	27.1	<0.1	<0.05	0.01	5.039	0.68	0.36	0.31
W6	2.83	6.38	200.5	334	28.1	0.3	<0.05	0.03	30.64	3.75	0.43	3.32
SW	4.61	6.9	182.9	311	29.1	0.3	<0.05	0.24	22.08	23.66	15.31	8.35

**Notes.**

DO, dissolved oxygen; ORP, oxidation-reduction potential; EC, Conductivity; Temp, temperature; TKN, Total Kjeldahl nitrogen; TP, total phosphorus; TC, total carbon.

### Genomic DNA extraction

All groundwater and surface water samples, approximately 1 liter for each sample, were filtered through a 0.2 µM membrane filter (Sigma-Aldrich, USA). Genomic DNA was extracted from the filter using FastDNA™ SPIN Kit for Soil (MP Biomedicals, USA), according to the manufacturer’s protocol. The extracted genomic DNA was quantified and verified using a NanoDrop spectrophotometer ND-100 (Thermo Fisher Scientific, USA) and agarose gel electrophoresis. It was then diluted to 5–10 ng µl^−1^ to use as a genomic DNA template for downstream analysis of the 16S rRNA and *aioA* genes

### 16S rRNA gene sequencing and data analysis

Extracted genomic DNA of 12 groundwater and one surface water samples was amplified, for each sample, in triplicate using a T100™ Thermal Cycler (Biorad, USA). The V3-V4 region of the 16S rRNA gene was amplified using previously published forward (5′-TCGTC

GGCAGCGTCAGATGTGTATAAGAGACAGCCTACGGGNGGCWGCAG-3′) and reverse primers (5′-GTCTCGTGGGCTCGGAGATGTGTATAAGAGACAGGACTA CHVGGGTATC TAATCC-3′) (Klindworth et al., 2013). Overhang adapter sequences of forward and reverse primers are 5′-TCGTCGGCAGCGTCAGATGTGTATAAGAGACAG-3′ and 5′-GTCTCGTG GGCTCGGAGATGTGTATAAGAGACAG-3′, respectively. The PCR mixture, with a total volume of 25 µl, was composed of 0.05 µl of each primer (100 mM), 0.5 µl of dNTPs (10 mM), 0.125 µl of Taq polymerase (New England Biolabs, USA), 2.5 µl of 10X ThermoPol reaction buffer, 1.5 µl of bovine serum albumin (BSA, 10 mg ml^−1^), and 1 µl of genomic DNA template. Amplification was conducted under the following conditions: 95 °C for 3 min, followed by 30 cycles at 95 °C for 30 s, 55 °C for 30 s, and 72 °C for 30 s, and a final extension at 72 °C for 5 min. Triplicate PCR products of each sample were pooled and purified using a NucleoSpin^®^ Gel and PCR Clean-up kit (Macherey-Nagel, Germany), following the manufacturer’s protocols. The quality and quantity of the purified PCR products were examined using the NanoDrop spectrophotometer ND-100 (Thermo Fisher Scientific, USA) and agarose gel electrophoresis. The purified PCR products were subsequently used for the Illumina library preparation using the MiSeq Reagent Kit V3, 500 cycles (2 × 250 bases; Illumina, USA), following the manufacturer’s protocol. Raw 16S rRNA gene amplicon sequence data are available in the Genbank database (SRA accession PRJNA630252).

During data analysis, raw amplicon sequences were evaluated using FastQC version 0.11.7. Forward and reverse primers were trimmed (17 and 21 bps, respectively) using Trimmomatic version 0.36. All processed sequences were applied to investigate the microbial profiles using Mothur 1.40.1 (Schloss et al., 2009) and following MiSeq SOP (https://mothur.org/wiki/miseq_sop/) with minor adjusted parameters and criteria specifically to the studied samples. The forward and reverse amplicon sequences were merged into contigs considering overlapped regions. These contigs were filtered using the criteria of sequence length between 430–470 bps, no ambiguous base and a maximum of 8 bps of homopolymer. Non-targeted region sequences were removed based on the reference database SILVA 132 (Quast et al., 2012). All candidate contigs were then de-noised and chimeric sequences were removed. Off-target sequences, including eukaryotes, chloroplast, and mitochondria, were also removed. *De novo* clustering was performed to identify operational taxonomic units (OTUs). Taxonomic assignment of the identified OTUs were based on the database SILVA 132. Alpha-diversity, including rarefaction curves, Chao1, and Shannon indices, was measured via Mothur. The numbers of reads in each sample were normalized by scaling based on the number of smallest total sequences of the investigated samples. Bray–Curtis dissimilarities were measured to compare the microbial community profiles and displayed via principal coordinates analysis (PCoA) and heatmap. Microbial compositions, PCoA and heatmap were plotted using in-house Python scripts. To investigate the relationship between microbial community structures and environmental factors, canonical correspondence analysis (CCA) was performed and plotted using the vegan R package (Dixon, 2003).

### *aioA* clone library preparation

The presence of the *aioA* gene in water samples was investigated using primers aoxBM1-2F-ND/aoxBM2-1R-ND (Quéméneur et al., 2010). The PCR mixture, with a total volume of 25 µl, contained 0.05 µl of each primer (100 µM), 0.5 µl of dNTPs (10 mM), 0.125 µl of Taq polymerase (New England Biolabs, USA), 2.5 µl of 10 × ThermoPol reaction buffer, 1.5 µl of BSA (10 mg ml^−1^), and 1 µl of genomic DNA template. To examine an optimal PCR condition for amplifying the *aioA* gene, a gradient annealing temperature function of 50−60 °C was performed using a T100™ Thermal Cycler (Biorad, USA). The PCR conditions started with an initial denaturation at 95 °C for 30 s, followed by 35 cycles at 95 °C for 30 s, 53−55 °C for 30 s, and 68 °C for 30 s, and a final extension at 68 °C for 5 min. Positive *aioA* amplified products were verified using agarose gel electrophoresis. Before *aioA* clone library construction, the *aioA* amplified products were purified using a NucleoSpin^®^ Gel and PCR Clean-up kit (Macherey-Nagel, Germany), according to the manufacturer’s protocols. Ligation and transformation were respectively conducted using pGEM^®^-T Easy Vector Systems (Promega, USA) and XL1-Blue supercompetent cells (Agilent, USA), following the manufacturer’s protocols. For each library, approximately 25 *aioA* clones were randomly selected for sequencing. The *aioA* sequences recovered from this study were submitted to GenBank (accession numbers MT432317 to MT432351).

### *aioA* -based phylogenetic construction

All retrieved *aioA* sequences were compared against those previously reported in the GenBank databases using blastn and blastx tools (Camacho et al., 2009). For each clone library, the *aioA* sequences were clustered into operational taxonomic units (OTUs) based on 3% cut-off using a CD-HIT program (Li & Godzik, 2006). Representative OTUs from each clone library were selected for phylogenetic analysis. All representative OTUs were aligned with selected reference cultured and uncultured *aioA* sequences using MUSCLE (Edgar, 2004). *Synechocystis* sp. was included as an outgroup. An *aioA*-based phylogenetic tree was generated using the MEGA package, version 7.0.21 (Kumar, Stecher & Tamura, 2016). A neighbor-joining tree was constructed using the maximum composite likelihood model with bootstrap values of 1,000 replicates (Tamura, Nei & Kumar, 2004).

### *aioA* gene quantification

The abundances of *aioA* and total 16S rRNA genes were estimated by quantitative PCR (qPCR) using a CFX96 real-time system (Bio-Rad, USA). Amplifications of the *aioA* and 16S rRNA genes were performed with primer sets aoxBM1-2F-ND/aoxBM2-1R-ND (Quéméneur et al., 2010) and 341f/518r (Muyzer, DeWaal & Uitterlinden, 1993), respectively. The relative abundance of *aioA* gene was expressed as the proportion of *aioA* to total bacterial 16S rRNA gene copies. The qPCR mixture contained 5 µl of SsoFast EvaGreen Supermix (Bio-Rad, Hercules, CA, USA), 0.03 µl of each primer (100 µM), 0.02 µl of BSA (10 mg ml^−1^), and 1 µl of DNA template (5 ng µl^−1^), in a total volume of 10 µl. The qPCR conditions started with an enzyme activation at 98 °C for 2 min, followed by 35 cycles of 98 °C for 5 s and 55 °C for 5 s, with a plate read after each cycle. However, the plate read was added at 84 °C to avoid the quantification of a primer dimer for the *aioA* gene quantification. After each run, melt curves were performed between 65–95 °C in 0.5 °C increments to verify the specificity of qPCR amplification. In addition, the specificity of qPCR products was checked by agarose gel electrophoresis. The standard curves of *aioA* and 16S rRNA amplifications were constructed from positive clones amplified by primer sets aoxBM1-2F-ND/aoxBM2-1R-ND and 341f/518r, respectively. The *aioA* and 16S rRNA PCR products were then purified using a NucleoSpin^®^ Gel and PCR Clean-up kit (Macherey-Nagel, Düren, Germany) and quantified by using a NanoDrop spectrophotometer ND-100 (Thermo Fisher Scientific, Waltham, MA, USA) to generate respective standard templates for qPCR. The qPCR standard curves were generated by ten-fold serial dilutions. An *aioA* standard curve was linear between 10^2^–10^7^ gene copies, with efficiencies of 93% (*R*^2^ = 1). A 16S rRNA standard curve was linear between 10^1^–10^7^ gene copies, with efficiencies of 102% (*R*^2^ = 0.998).

### Statistical analysis

A principal component analysis (PCA), based on the Euclidean distance, was calculated using MATLAB software (MathWorks, Natick, MA, USA) to investigate the similarity among the water samples collected from deep groundwater (DW), shallow groundwater (W), and surface water (SW). Correlations between each physicochemical factor were calculated using Pearson’s correlation coefficients and their corresponding *p*-values through MATLAB software. To identify physicochemical parameters significantly affecting the alpha diversity of microorganisms in water samples, the correlations between physicochemical parameters and alpha diversity indices were determined using Pearson’s correlation coefficients. The modified BIOENV method was also conducted to reveal a set of physicochemical parameters having the maximal Mantel correlations (Mantel, 1967) between Bray–Curtis and Gower distance matrices. The Bray–Curtis distance matrix was used to estimate dissimilarities between sites based on alpha diversity indices, while the Gower distance matrix was used to evaluate dissimilarities between sites based on physicochemical parameters. The BIOENV method is used to determine matrix correlation between the Bray–Curtis dissimilarity and the Euclidean distance matrices (Clarke & Ainsworth, 1993). In this study, the BIOENV method was modified by using the Gower distance matrix instead of the Euclidean distance matrix because it is more appropriate for our heterogeneous physicochemical parameters (Gower, 1971). To identify physicochemical parameters significantly affecting the community and abundance of *aioA* gene, the set of physicochemical parameters having the maximal Mantel correlations with the community and abundance of *aioA* gene were also determined using the modified BIOENV method. The Bray–Curtis distance matrix was used to determine dissimilarities based on the community and abundance of *aioA* gene, while the Gower distance matrix was used to evaluate dissimilarities between sites based on physicochemical parameters. All mentioned statistical analyses were performed using the Fathom Toolbox of MATLAB software (Jones, 2015).

## Results

### Water characteristics

**Figure 2:**
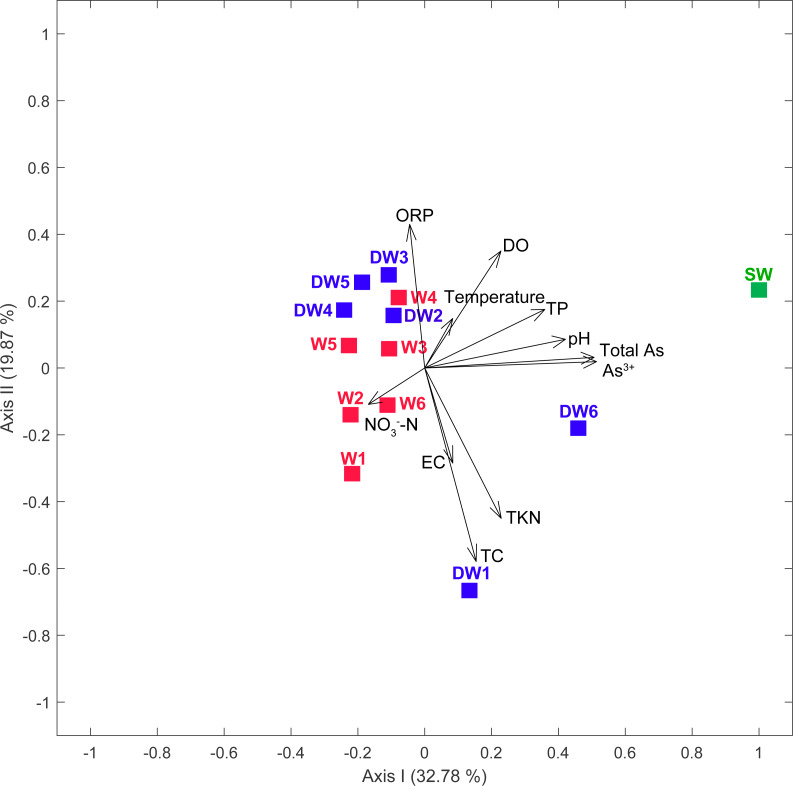
Principal component analysis (PCA) plot based on geochemical parameters of six deep groundwaters (DW1–DW6), six shallow groundwaters (W1–W6), and surface water (SW).

Groundwater samples, in total 12, were collected from 6 deep wells (tube wells) and 6 shallow wells (ring wells). One surface water sample was also collected from an old tailing pond in which the concentration of total arsenic was higher than the permissible limit of 10 µg l^−1^ recommended by WHO (Table 1). The concentration of total arsenic in surface water (SW) was 23.66 µg l^−1^, with the major species of As^3+^ (Table 1). The concentration of total arsenic in 12 groundwater samples ranged from 0.41 to 9.13 µg l^−1^, comprising As^3+^ (0.27 to 5.70 µg l^−1^) and As^5+^ (0.13 to 4.67 µg l^−1^). Temperatures and conductivity (EC) of water samples were 25.8 to 31.5 °C and 270 to 589 µs cm^−1^, respectively. Dissolved oxygen (DO) concentrations and pH ranged from 2.14 to 5.31 mg l^−1^ and 6.24 to 6.90, respectively (Table 1). Oxidation reduction potential (ORP) in all water samples was in the range of 64.8 to 217.2 mV, indicating slightly reducing to oxidation conditions. Total Kjeldahl Nitrogen (TKN) and NO_3_^−^-N concentrations were less than 0.1 to 0.4 mg l^−1^ and less than 0.05 to 0.12 mg l^−1^, respectively. The concentrations of total carbon (TC) across all samples were in a broad range of 1.66 to 51.66 mg l^−1^. A principal component analysis (PCA) showed that low concentrations of total phosphorus (TP), pH, total arsenic and As^3+^ typified water characteristics of the shallow groundwaters, while the high concentrations of these physicochemical parameters contributed to the distinct characteristics of surface water (Fig. 2).

### Alpha diversity of microorganisms in deep and shallow groundwaters, and surface water

Rarefaction curves demonstrated that the diversity richness of W1 was much higher than in the other samples (Fig. S2). The rarefaction curve of W1 did not reach an asymptote, indicating greater sequencing depth possibly leads to the detection of rare microbial taxa. Both the Chao1 and Shannon indexes also demonstrated that W1 harbored the most diverse microbial diversity although neither index demonstrated that W1 distinctly differed from the rest of the analyzed samples (Table S2). The rarefaction curves of the 12 samples, other than W1, were saturated, indicating optimal sequencing depth (Fig. S2). The diversity richness of all 12 samples (6 deep groundwater samples, 5 shallow groundwater samples, and 1 surface water sample) was comparable. The correlation between physicochemical parameters and alpha diversity indices of all water samples was investigated using Pearson’s correlation coefficient (Table S1). The microbial diversity in the analyzed water samples was positively correlated with TKN (*r* = 0.605, *p* = 0.029) and negatively correlated with temperature (*r* =  − 0.670, *p* = 0.012). The modified BIOENV method suggested that temperature, NO_3_^−^-N, and EC collectively shaped the alpha diversity of the microorganisms in the analyzed water samples (*r* = 0.515, *p* = 0.002). Overall, the results demonstrated that temperature, TKN, NO_3_^−^-N, and EC influenced the alpha diversity of microorganisms in deep groundwaters, shallow groundwaters, and surface water.

### Microbial community structures in deep and shallow groundwaters, and surface water

The 16S rRNA gene analysis showed that *Proteobacteria* were a major phylum, detected across all analyzed samples, accounting for 36–98% of the total microbial abundance (Fig. S3). Other microbial phyla highly represented in deep groundwaters, shallow groundwaters or surface water were *Cyanobacteria* (24%), *Actinobacteria* (31%), *Patescibacteria* (15%), *Bacteroidetes* (11%), and *Epsilonbacteraeota* (10%). Although these 5 detected phyla were highly abundant in particular samples, they were rare in the others (less than 0.01%), indicating the dynamics of microbial taxa across different aquifer types (Fig. S3).

**Figure 3:**
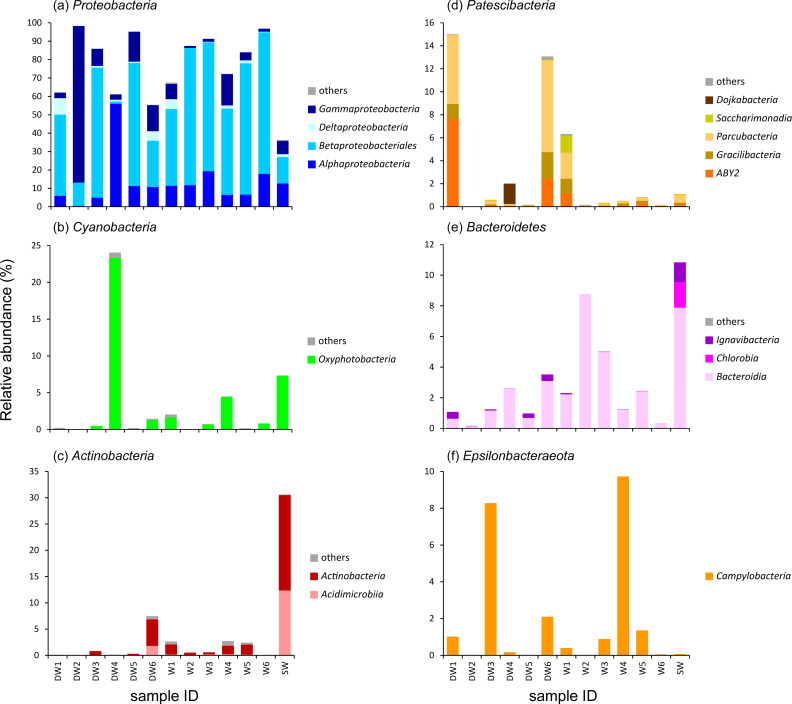
Relative abundance of microbial compositions at the class level of the six major phyla (A–F) found in six deep groundwaters (DW1–DW6), six shallow groundwaters (W1–W6), and surface water (SW). Only taxa with relative proportions >1% of the total microbial abundance in at least one sample are shown.

To better understand the microbial community structures in deep and shallow groundwaters, and surface water, the microbial abundances of the six dominant phyla were separately analyzed at the class level for comparison (Fig. 3). The phylum *Proteobacteria* found in the water samples was composed of four main classes: *Alphaproteobacteria*, *Betaproteobacteria*, *Deltaproteobacteria*, and *Gammaproteobacteria* which respectively showed their highest abundances in DW4 (56%), W6 (77%), DW1 (9%), and DW2 (85%) (Fig. 3A). *Betaproteobacteria* were the majority of microbial taxa detected in shallow groundwaters (42–77%). Although *Proteobacteria* were highly represented in both deep and shallow groundwaters, they were also present in surface water at lower abundance (Fig. 3A).

One major class belonging to the phylum *Cyanobacteria* found in our analyzed samples was *Oxyphotobacteria*, highly detected in DW4 (23%), SW (7%), W4 (4%), W1 (2%), and W6 (1%) (Fig. 3B). It was rare, however, (less than1%) in the other samples. The classes *Acidimicrobiia* and *Actinobacteria*, members of the phylum *Actinobacteria*, were highly detected in DW6 and SW (Fig. 3C). Although these two classes were not commonly detected in most deep groundwater samples, *Acidimicrobiia* and *Actinobacteria* were particularly found in DW6, accounting for 2% and 5% of the total microbial abundance, respectively. The class *Actinobacteria* was found as a minor assemblage across all shallow groundwaters. Surface water hosted high abundances of both *Acidimicrobiia* (12%) and *Actinobacteria* (18%). The abundance of the phylum *Patescibacteria* was relatively high in DW1 (15%), DW4 (2%), DW6 (13%), and W1 (6%), but barely detected in the other samples (Fig. 3D and Fig. S3). Three classes, ABY2, *Gracilibacteria*, and *Parcubacteria*, were highly represented in DW1, DW6, and W1, whereas the classes *Dojkabacteria* and *Saccharimonadia* were exclusively present in DW4 and W1, respectively (Fig. 3D). The class *Bacteroidia*, a member of the phylum *Bacteroidetes*, was commonly found at low abundance across all samples, ranging from less than 1% to 9% of the total microbial abundance (Fig. 3E). Surface water contained a high abundance of the phylum *Bacteroidetes* comprising the classes *Bacteroidia* (8%), *Chlorobia* (2%), and *Ignavibacteria* (1%). The class *Campylobacteria*, belonging to the phylum *Epsilonbacteraeota*, was highly represented in W4 (10%), DW3 (8%), DW6 (2%), W5 (1%), and DW1 (1%), while it was found at low abundance in the rest of the samples (less than 1%) (Fig. 3F).

**Figure 4:**
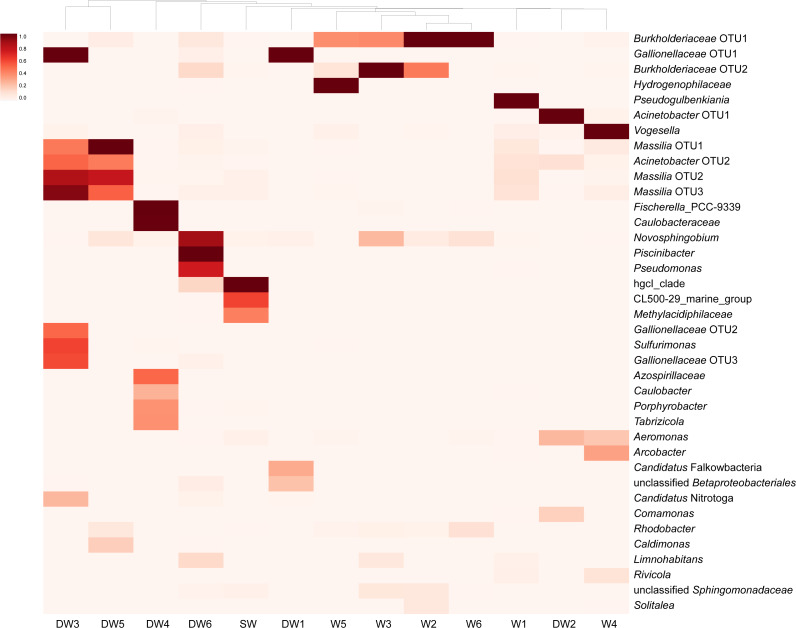
Heatmap based on the abundance of more than 3% OTUs. The relative proportions of microbial lineages are indicated by the color intensity.

A heatmap analysis, based on the presence of more than 3% OTU abundance, indicated the dominant microbial taxa in each sample (Fig. 4). The majority of *Betaproteobacteria* and Class ABY1 in DW1 were *Gallionellaceae* and *Candidatus* Falkowbacteria, respectively. DW2 was exclusively dominated by *Gammaproteobacteria* (85%) chiefly comprising the genera *Acinetobacter* and *Aeromonas*. *Betaproteobacteria* hosted by DW2 were mostly *Comamonas*. Unlike DW2, DW3 was primarily dominated by *Betaproteobacteria* (71%), mostly comprising *Massilia*, unclassified *Gallionellaceae*, and *Candidatus* Nitrotoga. The genus *Sulfurimonas*, a member of *Epsilonbacteraeota*, were also prevalent in DW3. DW4 was dominated by both uncultured *Caulobacteraceae* and *Fischerella* sp. PCC 9339, members of the classes *Alphaproteobacteria* and *Oxyphotobacteria*, respectively. Although DW5 was also dominated by *Betaproteobacteria* (67%), the dominant genera were *Massilia* and *Caldimonas* (Figs. 3A and 4). The dominant taxa found in DW6 were *Piscinibacter*, *Pseudomonas*, and *Novosphingobium*, members of the classes *Betaproteobacteria*, *Gammaproteobacteria*, and *Alphaproteobacteria*, respectively. Unlike the other deep groundwater samples, DW6 hosted a relatively high abundance of the *hgcI clade*, members of the phylum *Actinobacteria*.

As for shallow groundwaters, the heatmap analysis showed that W1 and W5 were respectively dominated by *Pseudogulbenkiania* and *Hydrogenophilaceae*, while *Burkholderiaceae* predominated in W2, W3, W5, and W6 (Fig. 4). These three taxa are members of the class *Betaproteobacteria*. Although W3 was dominated by *Burkholderiaceae*, *Novosphingobium* which are affiliated with the class *Alphaproteobacteria* were also highly detected. The dominant *Betaproteobacteria* found in W4 were the genus *Vogesella* and *Rivicola.* The genus *Arcobacter*, belonging to the class *Campylobacteria*, was also found in W4 at high abundance (Figs. 3F and 4). Like neither DW nor W, SW was dominated by *hgcI clade* and *CL500-29 marine group,* members of the class *Actinobacteria* and *Acidimicrobiia*, respectively.

### Factors influencing microbial community structures of deep and shallow groundwaters, and surface water

**Figure 5:**
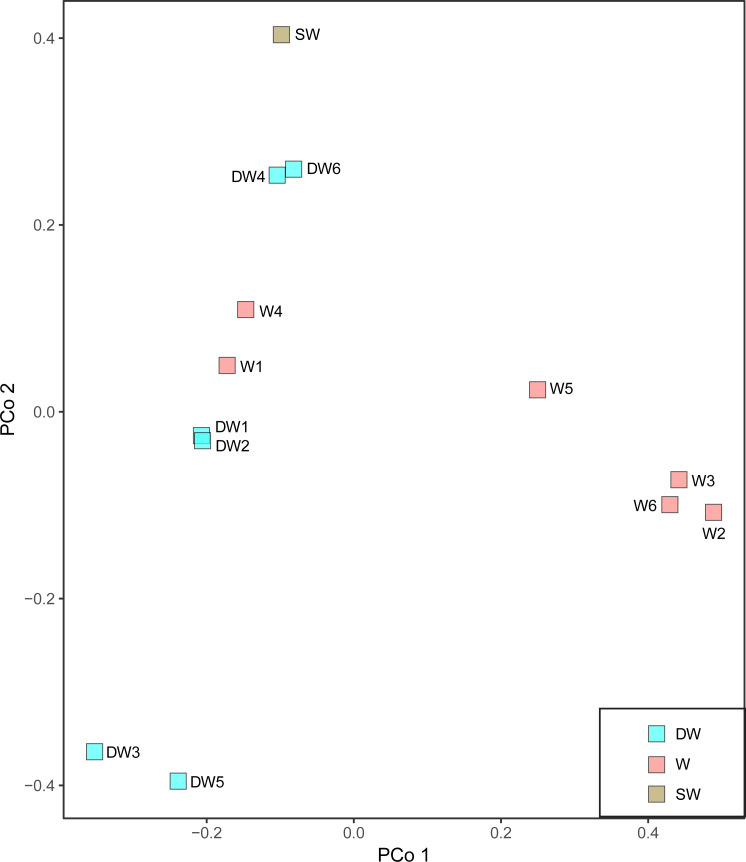
Principal coordinate analysis (PCoA) plot based on the Bray–Curtis dissimilarity matrix of microbial compositions in six deep groundwaters (DW1–DW6), six shallow groundwaters (W1–W6), and surface water (SW).

**Figure 6:**
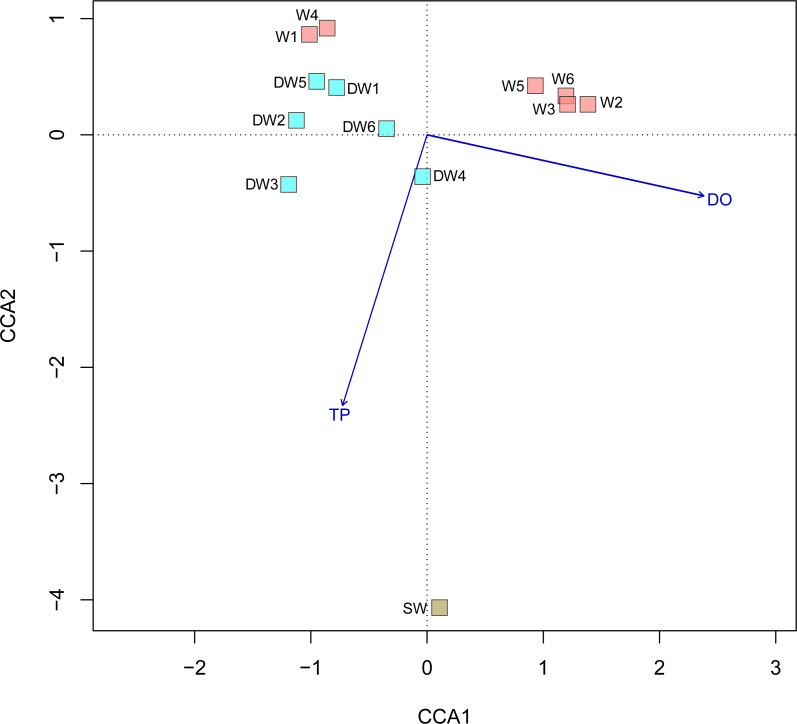
Canonical correspondence analysis (CCA) plot of microbial compositions and geochemical parameters (*p* < 0.05). Arrows indicate the correlation and magnitude of geochemical parameters associated with microbial community structures.

A principal coordinate (PCoA) analysis revealed that the microbial community structures in deep groundwater (DW), shallow groundwater (W), and surface water (SW) were different from one another (Fig. 5). A canonical correlation analysis (CCA) was also conducted to evaluate the relationship between physicochemical parameters and microbial community structures. The resulting CCA demonstrated that the concentrations of DO influenced the microbial community structure in most of the shallow groundwaters, while the low concentrations of TP were associated with the microbial community structure in the deep groundwaters (Fig. 6). The microbial community structure in surface water was influenced by the high concentrations of TP and DO.

**Figure 7:**
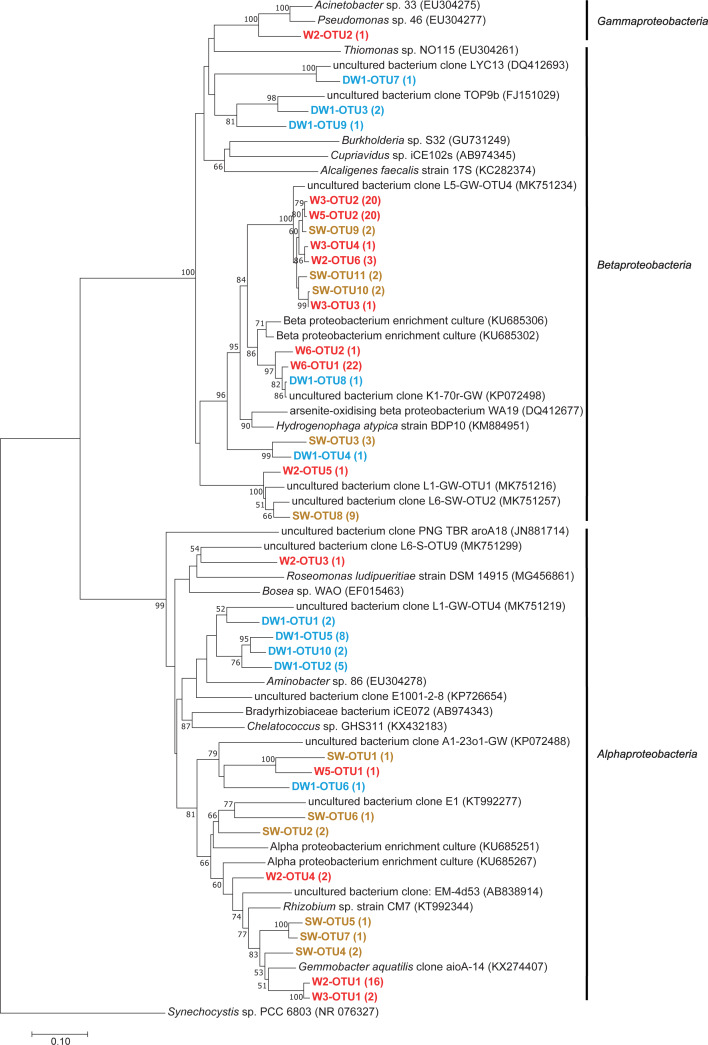
Neighbor-joining tree of partial nucleotide sequences of aioA gene retrieved from deep groundwater (DW), shallow groundwater (W), and surface water (SW). Samples are indicated in bold and the numbers of aioA sequences belonging to each OTU are indicated in parentheses. The bootstrap values >50% are shown.

### Diversity and abundance of the *aioA* genes in deep and shallow groundwaters, and surface water

A previous study reported that the arsenic concentration in groundwater from the study area was higher than the maximum admissible concentration of 10 µg l^−1^ (Tiankao & Chotpantarat, 2018). The current analysis of arsenic concentration in surface water showed a high concentration of arsenic which exceeded the standard limit (Table 1). In As-contaminated aquifers, arsenite-oxidizing bacteria play a crucial role in transforming highly toxic As^3+^ to less toxic As^5+^. Consequently, the diversity and abundance of arsenite-oxidizing bacteria in all water samples were investigated by analysis of large subunit of the functional gene arsenite oxidase (*aioA*) using PCR cloning-sequencing and qPCR. The *aioA* amplifications indicated that, six out of 13 samples (one deep groundwater, four shallow groundwaters, and one surface water) showed a positive signal. All positive *aioA* samples (DW1, W2, W3, W5, W6, and SW) were then cloned and sequenced. The results demonstrated that all analyzed *aioA* sequences were 99–100% identical to the protein arsenite oxidase and were 88–99% identical to previously reported *aioA* sequences retrieved from As-contaminated environments such as groundwater (Hassan et al., 2015), aquatic sediment (Yamamura et al., 2014), paddy soils (Hu et al., 2015), and biofilms elsewhere (Li et al., 2016; Osborne et al., 2010).

Phylogenetic analysis showed that the analyzed *aioA* sequences were associated with *Alphaproteobacteria*, *Betaproteobacteria*, and *Gammaproteobacteria* (Fig. 7). The *aioA*-based phylogenetic tree revealed two major branches with robust bootstrap values affiliated with *Alphaproteobacteria* and *Betaproteobacteria*. The majority of retrieved *aioA* sequences were affiliated with *Alphaproteobacteria* and *Betaproteobacteria*, while a gammaproteobacterial *aioA* sequence was found only in W2 at low abundance (Fig. 7 and Table 2). In deep groundwaters, *aioA* genes were only detected in DW1. The *aioA* sequences retrieved from DW1 were mainly grouped with *Alphaproteobacteria*, but those phylogenetically related to *Betaproteobacteria* were also discovered. The *aioA* genes were found in shallow groundwaters at higher frequency than in deep groundwaters. Most *aioA* sequences recovered from W3, W5, and W6 were associated with *Betaproteobacteria*, while those belonging to *Alphaproteobacteria* were a minor assemblage. The *aioA* sequences retrieved from W2 were mainly associated with *Alphaproteobacteria*, followed by *Betaproteobacteria* and *Gammaproteobacteria*. As for SW, the more *aioA* sequences were associated with *Betaproteobacteria* than with *Alphaproteobacteria* (Fig. 7 and Table 2).

The resulting qPCR demonstrated that the numbers of *aioA* and 16S rRNA genes were in the range of 3.7 ×  10^3^ ± 2.2 ×  10^2^ to 1.7 ×  10^5^ ± 4.8 ×  10^3^ and 4.3 ×  10^5^ ± 6.1 ×  10^4^ to 1.1 ×  10^6^ ± 8.2 ×  10^4^ copies per ng of genomic DNA, respectively (Table S3). The numbers of 16S rRNA gene copies were relatively consistent across all analyzed samples, indicating no bias caused by DNA extraction and different biomass. To better compare the abundance of *aioA* gene across all samples, the abundance of the *aioA* gene copies was normalized to that of the 16S rRNA gene copies. The relative abundance of the *aioA* gene found in water samples ranged from 0.85 to 37.13% (Table 2). To elucidate those physicochemical factors significantly affecting the diversity and abundance of *aioA* gene, a modified BIOENV was conducted. The results indicated that the combination of ORP and the concentration of NO_3_^−^-N influenced the diversity and abundance of *aioA* retrieved from this study (*r* = 0.521, *p* = 0.019).

**Table 2:** The relative abundances of alphaproteobacterial-, betaproteobacterial-, and gammaproteobacterial arsenite-oxidizing bacteria, and *aioA* gene copies detected in deep- (DW), shallow (W) groundwaters, and surface water (SW).

ID	arsenite-oxidizing bacteria			*aioA*/16S rRNA gene copies (%)
	*Alphaproteobacteria* (%)	*Betaproteobacteria* (%)	*Gammaproteobacteria* (%)
DW1	75	25	0	0.85
W2	79	17	4	3.60
W3	8	92	0	37.13
W5	5	95	0	1.98
W6	0	100	0	1.26
SW	31	69	0	5.18

## Discussion

Physicochemical characteristics of deep and shallow groundwaters were comparable. However, the concentrations of TP, total arsenic, and As^3+^ in deep and shallow groundwaters were lower compared to those of surface water. SW was collected from an old tailing pond where was surrounded by an intensively agricultural area. The elevated concentrations of TP, total arsenic, and As^3+^ in SW likely resulted from the effects of the old tailing pond and leaching of fertilizers and pesticides/herbicides. High As^3+^ concentration in SW may favor the presence of particular bacterial assemblages, especially arsenite-oxidizing bacteria, involved in mediating the arsenic cycle.

### Distinct microbial community structures in each aquifer

Rarefaction analysis suggested that, with deeper sequencing effort, rare microbial taxa were possibly discovered from W1, a shallow groundwater (Fig. S2). Previous studies have found that shallow aquifers hosted a higher diversity of microorganisms than deep aquifers (Lee, Unno & Ha, 2018; Sultana et al., 2011). However, the major phylum found in both deep and shallow groundwater microbiomes was *Proteobacteria* which comprised 55–98% of the total microbial abundance. Microbiome analysis revealed that *Proteobacteria* were the majority of groundwater microbiomes previously reported across different locations, including groundwater of Rayong Province, Thailand (37–93%; Sonthiphand et al., 2019), As-contaminated groundwater of Assam, India (63%, Das et al., 2017); groundwater of the Nakdong River Bank, South Korea (64–98%; Lee, Unno & Ha, 2018), and high As-contaminated groundwater in Northern Italy (∼70%, Cavalca et al., 2019).

DW2 and DW5 were exclusively dominated by *Proteobacteria* (Fig. 3A). The microbial structure of DW2 was mainly composed of *Acinetobacter*, *Aeromonas*, and *Comamonas*, while that of DW5 was heavily occupied by *Massilia* and *Caldimonas* (Fig. 4). *Acinetobacter* and *Aeromonas*, opportunistic pathogens, were isolated from South African groundwater aquifer affected by mining, agricultural, and municipal sewage (Carstens et al., 2014). *Acinetobacter* and *Aeromonas* were also dominant in groundwater from a thickly crowded market area in India (Patel et al., 2014). Agricultural and residential areas possibly contributed to the high abundance of *Acinetobacter* in groundwater of Rayong province, Thailand (Sonthiphand et al., 2019). In addition, *Acinetobacter* were commonly detected in As-contaminated groundwater where contributed to arsenic transformations (Das et al., 2016; Li et al., 2015a). *Massilia* were the dominant taxa found in As-contaminated groundwater of Hetao Basin in China (Li et al., 2013) and in a fermentation system, capable of the degradation of rice bran (Hou et al., 2019).

As with DW5, DW3 was dominated by *Massilia*: however, it also harbored other dominant members of the class *Betaproteobacteria*, such as *Gallionellaceae* and *Candidatus* Nitrotoga (Fig. 4). *Gallionellaceae* are a well-known iron oxidizer detected in groundwater. A metatranscriptomic analysis recently revealed their performance in nitrate reduction (Hassan et al., 2015; Jewell et al., 2016). *Candidatus* Nitrotoga, commonly known as nitrite-oxidizing bacteria, were present in both natural and engineered environments; metagenomic analysis indicated their versatile energy metabolisms involved in N, S, and C cycling (Boddicker & Mosier, 2018).

Three microbial taxa uniquely detected in DW4 at high abundance were *Fischerella* sp. PCC 9339, *Caulobacteraceae* and *Dojkabacteria*, members of the phyla *Proteobacteria*, *Cyanobacteria*, and *Patescibacteria*, respectively (Figs. 3 and 4). Although none of these was ubiquitous in groundwater environments, all have previously been detected in hot springs and in soils (Alcorta et al., 2018; Zhang et al., 2020). The phylum *Patescibacteria* was highly represented in DW1 and DW6. *Patescibacteria* are dominant in oligotrophic groundwaters as a result of their accumulation from soil microbiome leaching (Herrmann et al., 2019). The heatmap revealed that *Candidatus* Falkowbacteria were the dominant patescibacterial taxa found in DW1. Elsewhere they are not commonly found in groundwater; however, they have previously been detected in a thermokarst lake (Vigneron et al., 2020).

The genera *Piscinibacter*, *Novosphingobium*, and *Pseudomonas* constituted the majority of proteobacterial taxa found in DW6. Although *Piscinibacter* have been rarely documented in groundwater, a member of *Piscinibacter* was actively present in chloroethene-contaminated groundwater in the Czech Republic (Kotik et al., 2013). *Novosphingobium* were predominant in groundwater and they had ability to degrade organic pollutants (Tiirola et al., 2002). Like *Acinetobacter* and *Aeromonas*, *Pseudomonas* are opportunistic pathogens. *Pseudomonas* were commonly found in groundwater environments and dominated in groundwater impacted by sewage canals (Lee, Unno & Ha, 2018; Patel et al., 2014). The abundance of such opportunistic pathogens in groundwater may be used as an indicator of groundwater quality.

The majority of the microbial proportion found in the shallow groundwaters was *Betaproteobacteria* (42–77%) (Fig. 3A). W2, W3, W5, and W6 were mainly occupied by *Burkholderiaceae*. *Betaproteobacteria*, especially *Burkholderiaceae*, were abundant in As-contaminated groundwater and are potentially involved in As, Fe, and P cycling (Chakraborty, DasGupta & Bhadury, 2020; Hassan et al., 2015). The most abundant betaproteobacterial taxa found in W5, however, were *Hydrogenophilaceae* (Fig. 4), elsewhere found at high abundance in groundwater polluted by organic substances (Kotik et al., 2013). The major *Betaproteobacteria* detected in W1 were *Pseudogulbenkiania*, which are able to perform denitrification coupled with iron oxidation (Liu et al., 2018). Betaproteobacterial genera uniquely found at high abundance in W4 were *Vogesella* and *Rivicola* (Fig. 4). Although *Vogesella* and *Rivicola* were rarely found in groundwater at high abundance, these taxa were previously isolated from freshwater environments (Chen et al., 2015; Sheu et al., 2014).

Although surface water (SW) was dominated by *Proteobacteria* (36%), their abundance was lower than in the groundwaters (DW and W) (Fig. 3A). Unlike groundwater, surface water was mainly occupied by the *hgcI clade* and *CL500-29 marine group* which are members of the classes *Actinobacteria* and *Acidimicrobiia*, respectively (Figs. 3C and 4). Both taxa were found at high abundance in freshwater lakes and freshwater reservoirs (Keshri, Ram & Sime-Ngando, 2018; Ram, Keshri & Sime-Ngando, 2019).

Overall, the results suggested that although *Proteobacteria* were commonly detected in deep groundwaters, shallow groundwaters, and surface water, the dominant taxa found in each samples were likely unique. The combination of variable physicochemical conditions and unique features of each aquifer may contribute to distinctness of the microbial communities among different aquifers. The dominant taxa detected play critical roles in not only mediating the biogeochemical cycles (i.e., N, C, S, and As) but also degrading toxic compounds in aquatic environments. In addition, groundwater quality may be assessed by examining bacterial indicators, such as *Acinetobacter* and *Aeromonas*, and *Pseudomonas*.

The microbial community structures in deep groundwaters, shallow groundwaters, and surface water were likely unique within the same aquifer type (Fig. 5). A previous study reported that microbial community structures in unconfined and confined aquifers were distinguishable (Guo et al., 2019). Physicochemical parameters influencing the microbial community structures in the aquifers were the concentrations of DO and TP (Fig. 6). DO concentration and ORP primarily controlled the microbial communities in groundwaters collected from different depths (Lee, Unno & Ha, 2018). However, a study of groundwater in Luoyang area, China suggested that DO concentration showed no significant correlation with groundwater depth due to the complicating factors such as the groundwater conditions and prevailing land use (Li et al., 2015b). The elevated concentration of TP in surface water was possibly caused by agricultural run-off through fertilizer leaching (Masipan, Chotpantarat & Boonkaewwan, 2016; Worsfold, McKelvie & Monbet, 2016). Deep groundwaters were associated with low concentrations of TP because they were less likely to receive external contaminants, compared to surface water. In addition, the concentrations of TP were positively correlated with the concentrations of total arsenic and As^3+^ (Table S4). Previous studies suggested that application of phosphorus fertilizers led to high concentrations of arsenic in an impacted area and aquifers (Jayasumana et al., 2015; Lin et al., 2016).

### Diversity and abundance of arsenite-oxidizing bacteria in aquifers impacted by anthropogenic activities

Due to the water conditions and the history of the sampling site, the occurrence of arsenite-oxidizing bacteria was examined through analysis of the *aioA* gene. One deep groundwater sample, four shallow groundwater samples, and one surface water sample showed the presence of arsenite-oxidizing bacteria (Table 2). Shallow groundwaters and surface water were more sensitive to external disturbances (e.g., agricultural and mining activities) compared to deep aquifers, and hence provided more positive *aioA*. That said external inputs, including arsenic, NO_3_^−^, and organic substances, can be used as energy and carbon sources for promoting the growth of arsenite-oxidizing bacteria. Arsenite-oxidizing bacteria retrieved from this study belonged to *Alphaproteobacteria*, *Betaproteobacteria*, and *Gammaproteobacteria* (Fig. 7). Previous studies indicated the concurrence of alphaproteobacterial-, betaproteobacterial-, and gammaproteobacterial arsenite-oxidizing bacteria in aquifers, across different locations, impacted by a board range of arsenic concentrations (Cavalca et al., 2019; Hassan et al., 2015; Quéméneur et al., 2010). The relative abundances of *aioA* gene in the analyzed samples ranged from 0.85 to 37.13% (Table 2). The *aioA* gene copies were the most abundant in W3, followed by SW. The arsenic concentration in W3 used to be higher than 10 µg l^−1^, while that in the other shallow groundwaters was below 10 µg l^−1^ (Tiankao & Chotpantarat, 2018). High concentration of As^3+^ in SW possibly provided an energy source for arsenite-oxidizing bacteria. Long-term arsenic contamination would be expected to enhance the abundance of arsenite-oxidizing bacteria in the impacted aquifers. The samples (i.e., mat, sinter, and water) from geothermal areas, with the exception of one particular sample belonging to the highest temperature, harbored the *aioA* gene copies in the range of less than 0.10 to 19.50% (Jiang et al., 2014).

The analysis of *aioA* gene suggested that arsenite-oxidizing bacteria belonging to *Alphaproteobacteria*, *Betaproteobacteria*, and *Gammaproteobacteria* were present at low abundance, while the analysis of 16S rRNA gene revealed that *Alphaproteobacteria*, *Betaproteobacteria*, and *Gammaproteobacteria* were the major microbial assemblages found in the analyzed aquifers. Based on the analysis of 16S rRNA, the microbial taxa capable of arsenite oxidation were rarely identified. One possible explanation is that arsenite-oxidizing bacteria constitute a minor assemblage in groundwater and surface water microbiomes. Limitations of the16S rRNA database for taxonomic assignment of uncultured arsenite-oxidizing bacteria could be another explanation for unidentified arsenite-oxidizing bacteria through the 16S rRNA gene analysis. However, the heatmap analysis demonstrated that *Burkholderiaceae* were dominant in particular groundwaters (Fig. 4). A comprehensive study of *Burkholderiales* bacterial genomes revealed that their members harbor As-related genes, including the *aioA* gene (Li, Zhang & Wang, 2014). Another dominant taxon involved in the presence of arsenic in groundwater is *Gallionellaceae*. Members of the *Gallionellaceae*, well-known iron-oxidizing bacteria, are able to produce iron oxides which subsequently adsorb arsenic in groundwater. The co-dominance of *Burkholderiaceae* and *Gallionellaceae* has the potential to impact arsenic immobilization in groundwater. A previous study also suggested that *Betaproteobacteria*, including *Burkholderiaceae* and *Gallionellaceae*, played a role in mediating arsenic cycling in As-contaminated groundwater (Chakraborty, DasGupta & Bhadury, 2020).

The diversity and abundance of arsenite-oxidizing bacteria retrieved from this study were affected by the combination of ORP and the concentration of NO_3_^−^-N (*r* = 0.521, *p* = 0.019). Previous study also showed the effect of NO_3_^−^-N concentration on groundwater microbial communities (Ben Maamar et al., 2015). In environments under reducing conditions, arsenite-oxidizing bacteria are able to anaerobically oxidize As^3+^to As^5+^, using As^3+^ as an electron donor and NO_3_^−^ as an electron acceptor. Sources of NO_3_^−^-N groundwater contaminations, analyzed by isotopic signatures, were soil organic nitrogen, fertilizer leaching, and manure/household waste (Nikolenko et al., 2018). Addition of NO_3_^−^ enhanced the abundance of *aioA* gene and stimulated the activity of anaerobic arsenite-oxidizing bacteria in flooded paddy soils and a laboratory-scale reactor (Sun et al., 2009; Zhang et al., 2017).

## Conclusion

The microbial community structures in deep and shallow groundwaters from an agricultural area were examined through the analysis of 16S rRNA and *aioA* genes. Surface water from the old tailing pond within the same locality of the groundwater sampling site was also included in the analysis. Microbial community structures were likely distributed according to the aquifer types, resulting from different physicochemical properties and hydrogeological characteristics of each aquifer type. In addition to the aquifer types, the microbial community structures in deep groundwaters, shallow groundwaters, and surface water were influenced by the concentrations of DO and TP. Consequently, both geological and physicochemical factors shaped the microbial community structures in the analyzed aquifers. Dominant taxa found in the analyzed aquifers appeared to be unique. They play crucial roles in mediating biogeochemical cycles (e.g., N, C, As, and Fe) and in degrading toxic substances. The co-dominance of *Burkholderiaceae* and *Gallionellaceae* potentially controlled arsenic immobilization in groundwaters. Analysis of the *aioA* gene suggested that arsenite-oxidizing bacteria were found at higher frequency in the shallow aquifers. The arsenite-oxidizing bacteria recovered from this study were associated with *Alphaproteobacteria*, *Betaproteobacteria*, and *Gammaproteobacteria*. External inputs from anthropogenic activities, especially through ferlilizer leaching, and aquifer conditions may enhance the abundance and activity of anaerobic arsenite-oxidizing bacteria. This study provides insights into microbiomes in deep and shallow aquifers, including surface water, and suggests further exploration of gene expression within groundwater, representing a unique microbial niche, using shotgun metatranscriptomic analysis.

##  Supplemental Information

10.7717/peerj.10653/supp-1 Supplemental Information 1 Supplemental Figures and Tables

## Additional Information and Declarations

### Field Study Permissions

The following information was supplied relating to field study approvals (i.e., approving body and any reference numbers): Mr. Narong Ketprapum, the President of Dan Chang Subdistrict Administrative Organization, and Mr. Surasi Songcharoen, the President of Nong Prue Subdistrict Administrative Organization, gave verbal permission for the collection of water samples.

### Data Availability

The following information was supplied regarding data availability: Raw 16S rRNA gene amplicon sequence data are available in Genbank: PRJNA630252. The aioA sequences are also available in GenBank: MT432317 to MT432351.
