# Supplementary material for: Microbial community structure in aquifers associated with arsenic: analysis of 16S rRNA and arsenite oxidase genes
Source: PeerJ. 2021 Jan 8;9:e10653. doi: 10.7717/peerj.10653 (PMC7798605; doi:10.7717/peerj.10653)
Supplement: Supplemental Information 1 [file peerj-09-10653-s001.docx]

**Supplementary Information**

**Microbial community structure in aquifers associated with arsenic: analysis of 16S rRNA and arsenite oxidase genes**

Prinpida Sonthiphand^1*^, Pasunun Rattanaroongrot^1^, Kasarnchon Mek-yong^1^, Kanthida Kusonmano^2,3^, Chalida Rangsiwutisak^2^, Pichahpuk Uthaipaisanwong^2^, Srilert Chotpantarat^4,5,6^, Teerasit Termsaithong^7,8^

^1^Department of Biology, Faculty of Science, Mahidol University, Bangkok, Thailand

^2^Bioinformatics and Systems Biology Program, School of Bioresources and Technology, King Mongkut’s University of Technology Thonburi, Bang Khun Thian, Bangkok, Thailand

^3^Systems Biology and Bioinformatics Research Laboratory, Pilot Plant Development and Training Institute, King Mongkut’s University of Technology Thonburi, Bangkok, Thailand

^4^Department of Geology, Faculty of Science, Chulalongkorn University, Bangkok, Thailand

^5^Research Program on Controls of Hazardous Contaminants in Raw Water Resources for Water Scarcity Resilience, Center of Excellence on Hazardous Substance Management (HSM), Chulalongkorn University, Bangkok, Thailand

^6^Research Unit of Green Mining (GMM), Chulalongkorn University, Bangkok, Thailand

^7^Learning Institute, King Mongkut’s University of Technology Thonburi, Bangkok, Thailand

^8^Theoretical and Computational Science Center (TaCS), King Mongkut’s University of Technology Thonburi, Bangkok, Thailand

*To whom correspondence should be addressed.

E-mail: prinpida.son@mahidol.ac.th

Phone: (+66) 2201-5250

Fax: (+66) 2354-7161

a) b)


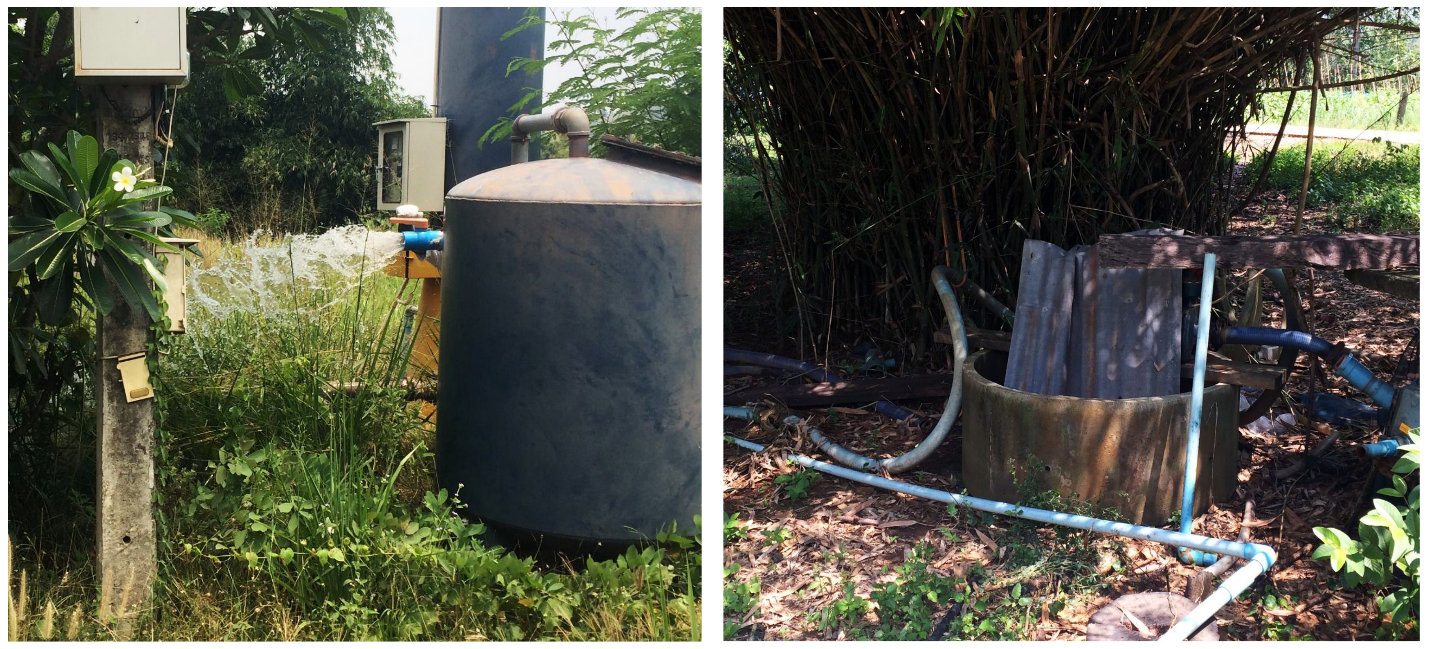


Fig. S1. a) Deep groundwater (tube well) and b) shallow groundwater (ring well)


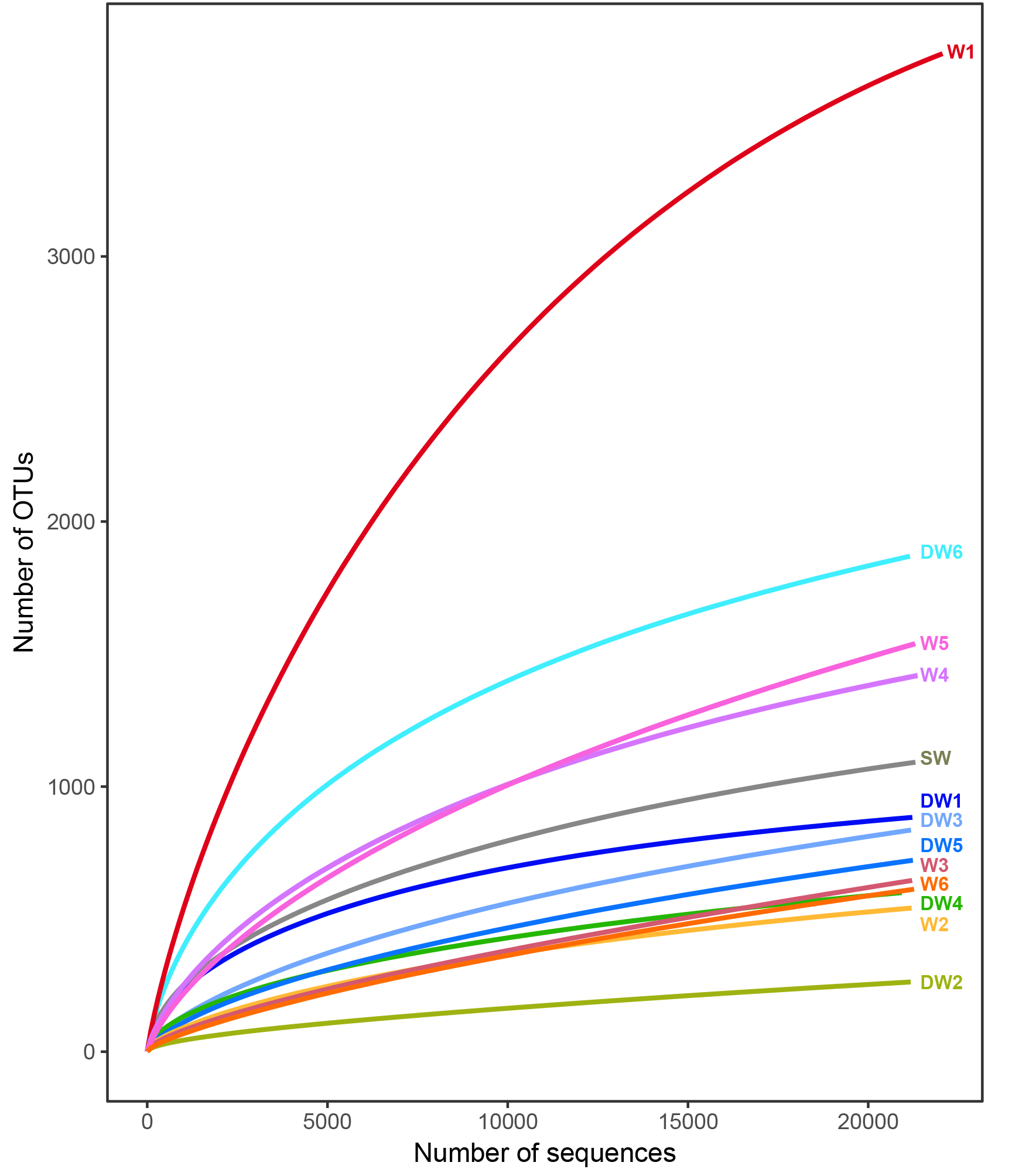


Fig. S2. Rarefaction curves based on the number of OTUs of the bacterial 16S rRNA gene in six deep groundwaters (DW1-DW6), six shallow groundwaters (W1-W6), and surface water (SW).


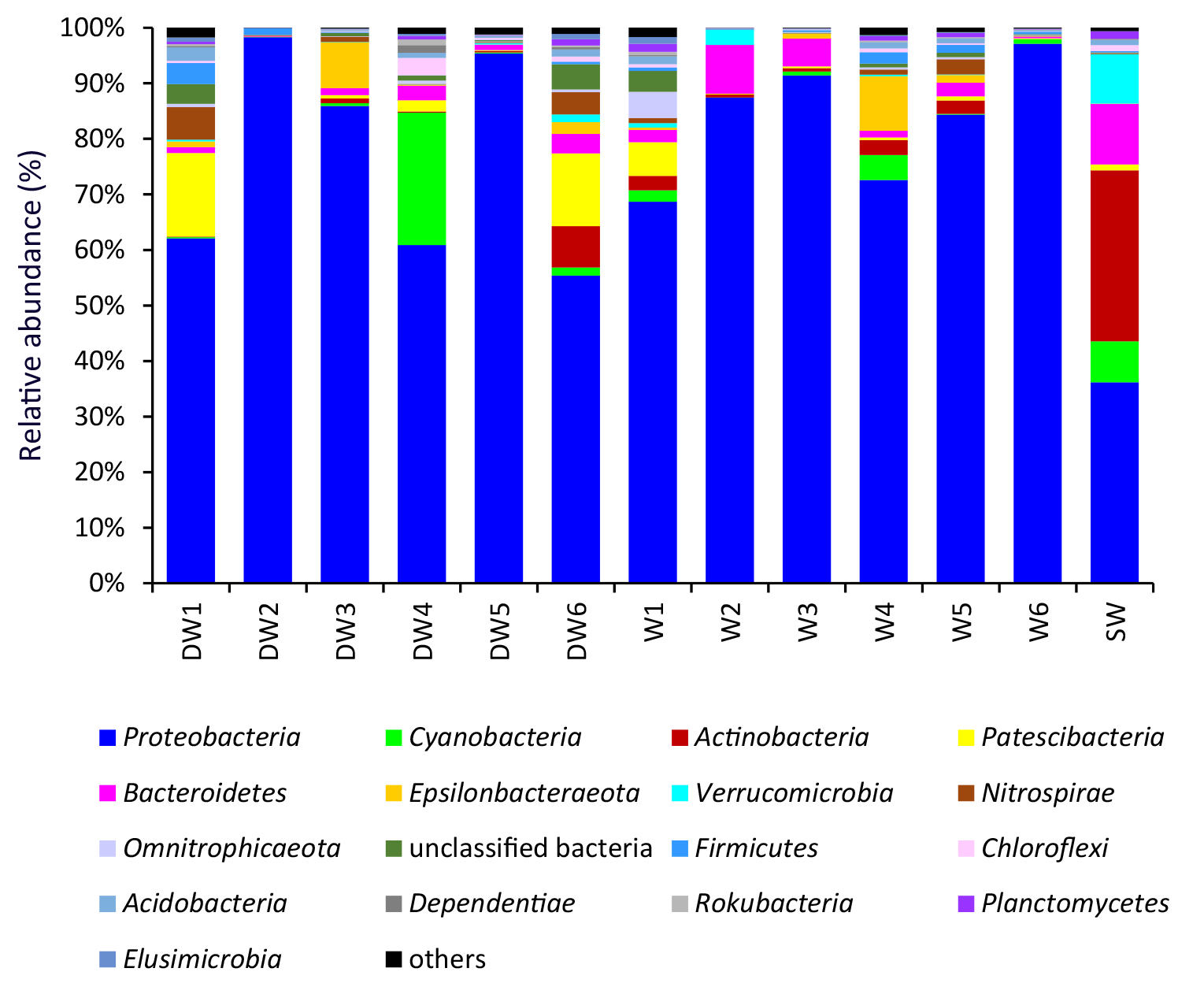


Fig. S3. Relative abundance of microbial compositions at the phylum level in deep groundwater (DW), shallow groundwater (W), and surface water (SW)

Table S1. Summary of diversity indices

| ID | Diversity indices | |
| --- | --- | --- |
|  | Chao1 | Shannon |
| DW1 | 1199.75 | 4.57 |
| DW2 | 704.77 | 1.66 |
| DW3 | 1484.40 | 3.56 |
| DW4 | 906.01 | 3.59 |
| DW5 | 1329.72 | 2.96 |
| DW6 | 2590.69 | 5.85 |
| W1 | 4307.03 | 6.45 |
| W2 | 860.13 | 2.53 |
| W3 | 2324.33 | 2.37 |
| W4 | 2096.52 | 4.22 |
| W5 | 3109.16 | 3.42 |
| W6 | 1485.85 | 1.56 |
| SW | 1455.24 | 4.89 |

Table S2. Pearson’s correlation coefficients (*r*) between geochemical parameters and alpha diversity indices

| Alpha diversity indices | Geochemical parameters | | | | | | | | | | |
| --- | --- | --- | --- | --- | --- | --- | --- | --- | --- | --- | --- |
|  | DO  (mg l^-1^) | pH | ORP (mV) | EC  (µs cm^-1^) | Temp (°C) | TKN (mg l^-1^) | NO_3_^-^-N (mg l^-1^) | TP  (mg l^-1^) | TC  (mg l^-1^) | Total As (µg l^-1^) | As^3+^ (µg l^-1^) |
| Chao1 | -0.201 | -0.010 | 0.046 | 0.060 | **-0.670** | 0.351 | 0.006 | -0.055 | 0.014 | -0.095 | -0.079 |
| Shannon | 0.044 | 0.491 | -0.176 | 0.207 | -0.438 | **0.605** | -0.093 | 0.068 | 0.234 | 0.339 | 0.419 |

Signiﬁcant differences (p < 0.05) are shown in bold.

Table S3. The abundances of *aioA* and 16S rRNA genes estimated by qPCR

| ID | Abundance of *aioA* gene  (copies per ng of DNA) | Abundance of 16S rRNA gene  (copies per ng of DNA) |
| --- | --- | --- |
| DW1 | 3.7x10^3^±2.2x10^2^ | 4.3x10^5^±6.1x10^4^ |
| W2 | 3.8x10^4^±3.1x10^3^ | 1.1x10^6^±8.2x10^4^ |
| W3 | 1.7x10^5^±4.8x10^3^ | 4.5x10^5^±1.5x10^4^ |
| W5 | 1.3x10^4^±9.2x10^2^ | 6.4x10^5^±6.3x10^4^ |
| W6 | 1.0x10^4^±3.0x10^2^ | 8.0x10^5^±1.3x10^5^ |
| SW | 2.9x10^4^±7.3x10^2^ | 5.6x10^5^±1.1x10^5^ |

Table S4. Pearson’s correlation coefficients (*r*) between each geochemical parameter

|  | DO  (mg l^-1^) | pH | ORP  (mV) | EC  (µs cm^-1^) | Temp  (°C) | TKN (mg l^-1^) | NO_3_^-^-N (mg l^-1^) | TP  (mg l^-1^) | TC  (mg l^-1^) | Total As (µg l^-1^) | As3^+^ (µg l^-1^) |
| --- | --- | --- | --- | --- | --- | --- | --- | --- | --- | --- | --- |
| DO  (mg l^-1^) | 1.00 | **0.70** | 0.30 | -0.05 | 0.36 | -0.10 | 0.04 | 0.09 | -0.16 | 0.26 | 0.34 |
| pH | **0.70** | 1.00 | 0.07 | 0.35 | 0.10 | 0.28 | -0.26 | 0.23 | 0.12 | **0.64** | **0.73** |
| ORP  (mV) | 0.30 | 0.07 | 1.00 | -0.32 | -0.06 | -0.09 | 0.18 | 0.06 | -0.50 | -0.08 | -0.07 |
| EC  (µs cm^-1^) | -0.05 | 0.35 | -0.32 | 1.00 | 0.15 | 0.11 | -0.34 | -0.38 | 0.28 | 0.03 | 0.10 |
| Temp  (°C) | 0.36 | 0.10 | -0.06 | 0.15 | 1.00 | -0.30 | 0.07 | 0.08 | 0.07 | 0.13 | 0.10 |
| TKN (mg/l) | -0.10 | 0.28 | -0.09 | 0.11 | -0.30 | 1.00 | 0.19 | 0.13 | **0.74** | 0.37 | 0.39 |
| NO_3_^-^-N (mg l^-1^) | 0.04 | -0.26 | 0.18 | -0.34 | 0.07 | 0.19 | 1.00 | -0.16 | 0.30 | -0.37 | -0.29 |
| TP  (mg l^-1^) | 0.09 | 0.23 | 0.06 | -0.38 | 0.08 | 0.13 | -0.16 | 1.00 | -0.02 | **0.82** | **0.74** |
| TC  (mg l^-1^) | -0.16 | 0.12 | -0.50 | 0.28 | 0.07 | **0.74** | 0.30 | -0.02 | 1.00 | 0.21 | 0.23 |
| Total As (µg l^-1^) | 0.26 | **0.64** | -0.08 | 0.03 | 0.13 | 0.37 | -0.37 | **0.82** | 0.21 | 1.00 | **0.98** |
| As3^+^  (µg l^-1^) | 0.34 | **0.73** | -0.07 | 0.10 | 0.10 | 0.39 | -0.29 | **0.74** | 0.23 | **0.98** | 1.00 |

Signiﬁcant differences (*p* < 0.05) are shown in bold.
